# Unpacking Cigar Product Familiarity and Terminology among Black and Hispanic Youth: It’s All about Blunts

**DOI:** 10.3390/ijerph19031689

**Published:** 2022-02-01

**Authors:** Dawnyéa D. Jackson, Emily C. Sanders, Molly Barry, Dana E. Wagner, Megan Wall Vigorita, Mario A. Navarro

**Affiliations:** 1 Rescue Agency, 660 Pennsylvania Avenue SE, Suite 400, Washington, DC 20003, USA; mbarry@rescueagency.com (M.B.); dana@rescueagency.com (D.E.W.); 2 The U.S. Food and Drug Administration, Center for Tobacco Products, 10903 New Hampshire Avenue, Silver Spring, MD 20993, USA; emily.sanders@fda.hhs.gov (E.C.S.); megan.wall@fda.hhs.gov (M.W.V.); mario.navarro@fda.hhs.gov (M.A.N.)

**Keywords:** cigars, little cigars, cigarillos, youth, terminology, tobacco, marijuana, tobacco education, public health, health communications

## Abstract

The prevalence rate of Cigar, Little Cigar, and Cigarillo (CLCC) use among youth rose above the rate of cigarettes for the first time in 2019, and Black and Hispanic youth remain disproportionately more susceptible and likely to use these products compared to White youth. Addressing this disparity through education requires a clearer understanding of the ways youth refer to, and group, CLCCs. Twenty-eight virtual focus groups with youth ages 13–17 (*n* = 105) were conducted across the U.S. Groups were split by race/ethnicity, with 14 Black and 14 Hispanic groups, and further divided by CLCC experimenters and susceptible users. Youth participants discussed CLCC use behaviors, harm and risk perceptions, and knowledge, attitudes, and beliefs about CLCC products. When shown photos of CLCC products during focus groups, youth across groups identified and labeled these products into four subcategories. Large cigars were universally labeled “cigars”. Little cigars were unfamiliar and guessed to be cigarettes. Tipped cigarillos were synonymous with the brand Black and Mild and considered tobacco. Untipped cigarillos were most referred to as “blunts” and used with marijuana. Understanding the nuances of language and use patterns of CLCCs is critical to ensuring the accuracy of measurement and impact of public health communications.

## 1. Introduction

In 2019, for the first time, the National Youth Tobacco Survey (NYTS) reported that the prevalence rate of Cigar, Little Cigar, and Cigarillo (CLCC) use among youth (Youth is a range of ages from 9–19 depending on the research cited. Please reference each article for specific age ranges.) (5.3%) rose above the rate of cigarette use (4.3%; [1]). The trend continued in 2020 (3.5% for CLCCs, 3.3% for cigarettes) making CLCCs the second most-used tobacco product for the second year in a row [2]. While the rates are still substantially lower than self-reported e-cigarette use (20% in 2019; 13.1% in 2020), it is notable that there is also a persistent and well-documented trend of disproportionate use of CLCCs among youth of color [1,2]. Current CLCC use rates are higher among Black (6.5%) and Hispanic (4.0%) youth compared to White youth (2.8%) and the overall sample (3.5%; [2]). Furthermore, CLCC products are heavily marketed and priced lower in minority communities, which increases the risk for minority youth exposure and use [3,4]. Addressing this important public health issue requires an understanding of the factors that lead to greater susceptibility and use.

### 1.1. CLCC Terminology and Measurement

The U.S. Food and Drug Administration (FDA) defines CLCCs as a category encompassing a diverse range of combustible tobacco products that are either wrapped in a tobacco leaf or paper that contains tobacco or tobacco extract [5]. Large or premium cigars are hand rolled with a whole tobacco leaf wrapper [6]. Little cigars resemble cigarettes but are wrapped in brown paper that contains some tobacco leaf and have a paper filter like a cigarette [5]. Cigarillos consist of tobacco wrapped in dried tobacco leaf or in any substance containing tobacco. Cigarillos are available in many flavors, and with or without a filter tip. While terminology of individual products has been standardized within the scientific community, there remains inconsistency in how this terminology is used and understood by the general public [7].

Specifically, there is evidence that product terminology used by researchers does not match terminology used by CLCC product users, leading to potential errors in self-report data. Both adults and youth consider the term “cigar” to only refer to large cigars while cigarillo and little cigar products are referred to by brand names and/or slang terms [5,8]. A recently published study found that youth and young adults had varied understandings of the term cigarillo [9]. Yerger et al. [8] asked participants about ever cigar use before and after focus groups and found that 12% more focus group participants reported ever cigar use after the focus group than before. This suggests they may have learned that a tobacco product they referred to by a different name could be considered a cigar. Adding brand names as examples in CLCC use questions in surveys has consistently been shown to increase reporting of youth cigar use rates compared to when brand names are not used [10]. In one national study, reported prevalence of CLCC use among Black youth increased from 19.5% in 2011 to 27.8% in 2012 when the question changed to include CLCC brand examples [11].

Additionally, current national surveys, such as NYTS, predominantly measure use and susceptibility to CLCCs as a broad category while other surveillance instruments examine only certain products (e.g., cigarillos), which does not account for the diversity of products. These measurement inconsistencies across scientific studies present barriers to understanding use patterns by product.

### 1.2. Blunt Terminology and Measurement

A compounding challenge to both measurement of prevalence rates and user terminology of CLCC products is the prominence of marijuana and CLCC co-use among youth. Large-scale studies report that modification of CLCCs, and untipped cigarillos specifically, for use as blunts (tobacco leaf products that have been emptied of some or all their tobacco and refilled with marijuana) is common among youth, with disproportionate use by both Black and Hispanic youth in urban areas [12,13]. Blunts are so commonly associated with untipped cigarillos, one study found that Black young adult (Young adult is a range of ages from 18–32 depending on the research cited. Please reference each article for specific age ranges) cigarillo users overwhelmingly thought that cigarillo companies designed their products and packaging to make the blunt-making process simple and enjoyable [14]. Indeed, certain companies have developed CLCC products that facilitate blunt making such as perforated lines or easily unrolled tobacco leaf, and resealable pouches for discretely storing assembled blunts [14]. Studies on cigarillo and blunt use demonstrate low familiarity with cigarillos as a tobacco product and high knowledge about blunts among youth and young adults, with untipped cigarillos reported as rarely being used as intended for tobacco consumption [10,14,15,16,17,18,19,20]. With this evidence in mind, it is important to further explore the ways youth most at risk of CLCC use understand and describe them, to disentangle which products are being used and how.

### 1.3. Current Study

These inconsistencies in product terminology underscore the importance of highlighting terms that are used by intended audiences and using culturally appropriate language in research. Previous research on CLCC terminology and use patterns has mostly focused on Black young adults and adults (most notably Dickinson and colleagues’ 2016 paper on cigar product terminology used by adults) with scant literature available on youth [6]. Additionally, although use of CLCCs among Hispanic youth is higher than overall use in the U.S., research on CLCC terminology and use patterns among this audience has been negligible. In this study we examine terminology and use patterns for CLCC products among a geographically diverse sample of both Black and Hispanic youth to understand which products are used, how they are consumed, and the terminology used to describe them. Understanding the terminology commonly used by youth and the prevalence patterns of youth CLCC susceptibility and use will aid in the development of more effective prevention messaging and ultimately more successful public health strategies and programs.

## 2. Materials and Methods

Self-identified Black and Hispanic participants, ages 13–17, were recruited using a panel recruitment agency. Focus groups were sourced from four U.S. regions (West, Southeast, Midwest, Northeast) with an emphasis on recruiting participants from the following city hubs and environs: Los Angeles, Atlanta, Chicago, and the District of Columbia. Focus groups were conducted in June and July of 2020. Since this study was conducted during the COVID-19 pandemic, all focus groups were conducted online, with participants engaging in a place where they could be given privacy and maintain physical distance and safety. This study was approved by Advarra’s Institutional Review Board.

### 2.1. Screening Criteria

Participants were verbally screened by age, race/ethnicity, and CLCC use status. Focus groups were segmented by race/ethnicity, CLCC use status, and location. Participants that identified as both Black and Hispanic were given the option to participate in the racial/ethnic focus group assignment that felt most comfortable. Based on responses to CLCC use items, participants were separated into focus groups of Experimenters (i.e., individuals who indicated that they have tried a CLCC product) or Susceptible Non-Triers (i.e., individuals who responded that they have never tried CLCC products but did not answer “definitely not” to all the items in an adapted Pierce susceptibility scale [21]. The Experimenter item was: “Have you ever tried cigars, cigarillos, or little cigars, even one or two puffs?” Susceptibility items included: “Do you think you will smoke a cigar, cigarillo, or little cigar in the next year?”; “Do you think you will smoke a cigar, cigarillo, or little cigar soon?”; “If one of your best friends were to offer you a cigar, cigarillo, or little cigar, would you smoke it?”. Response options ranged from “definitely not” to “definitely yes”. Questions used to assess CLCC status included example brand names to further assist participants in understanding the product of interest. Frequency of CLCC use was not assessed during screening. See demographic breakdowns in Table 1.

Based on early findings from the first two focus groups around lack of familiarity with the term cigarillo and the organic discussions around pervasive use of cigarillos as blunts (as described in the findings section below) the research team updated the eligibility questions in the screener to ensure that measures for use and susceptibility to CLCC products explicitly asked about using these products without marijuana. Participants who reported susceptibility to or experimentation with CLCC products without marijuana qualified for the study. Blunt use was not assessed during screening. Ever use and susceptibility of e-cigarettes was also measured on the screener but was not a qualification for participation.

### 2.2. Focus Group Procedure

Immediately before logging on to the focus group, participants completed an online check-in survey. Focus groups were 90 min in length and conducted on Zoom Webinar. Only the moderator was able to be viewed via their webcam. All participants and observers joined via audio only due to privacy concerns from the Institutional Review Board, given that youth participants self-reported the use of illegal tobacco and marijuana products on surveys and discussed youth product use. Participants were made aware of other focus group observers but did not have the ability to privately communicate with or see observers. The moderator began with an icebreaker activity to build rapport and comfort with the platform and format. This was followed by general questions about everyday activities (to understand participants lived experiences) including how these have changed considering the COVID-19 pandemic. Next, the discussion focused on youth CLCC product use and perceptions (which lasted approximately 40 min) and is the focus of the present paper. Following this section, the moderator tested a series of statements or facts related to CLCC products to gather opinions and sentiments. For more information on the guide, please see the Measures section.

### 2.3. Check-In Survey Measures

The 13-question Check-In Survey, administered immediately before the group discussion, included measures of tobacco and marijuana use, norms, perceived harm and addiction, and adverse childhood experiences. Participants were emailed a unique online check-in survey link and given instructions to complete the survey prior to joining the focus group. For the current study, only three items related to tobacco and marijuana use were analyzed: “Have you ever tried cigarette smoking, even one or two puffs?”; “Have you ever tried smoking a blunt (cigar, cigarillo, or little cigar) with marijuana (pot, weed, or cannabis) even one or two puffs?”; and “Have you ever tried using THC (marijuana) as a liquid in an electronic cigarette or vape pen, even one or two puffs?”. Responses options included “Yes” or “No”.

### 2.4. Focus Group Moderator’s Guide

The moderator guide comprised a series of questions and probes to understand the terminology and groupings of various CLCC products, reasons for use, health risk perceptions, and perceptions of relative risk and harm compared to other tobacco products. Additionally, the guide contained specific questions and probes on the relationship between CLCCs and marijuana.

### 2.5. Data Analyses

Quantitative analyses were performed using SPSS and qualitative analyses were manually performed using Excel. Screener and Check-In Survey data were entered, cleaned, and checked for quality control. The de-identified data file was imported into SPSS for analysis, and indicator variables were constructed as needed. Descriptive analyses (frequencies and percentages) were conducted (see Table 1). Each of the focus groups were audio-recorded, with the permission of participants, and transcribed. The lead author developed a data extraction tool created in Excel. Sections of the moderator guide were then categorized based on emergent themes (e.g., CLCC Product Familiarity, Terminology, Use Trends, Harm and Addiction Perceptions of Tobacco and Marijuana, etc.). Following a training led by the lead author, coders were assigned a discussion topic or set of probes to review in each transcript. Coders then developed a series of statements to describe the patterns of responses and differences between subgroups, when relevant. The lead author then reviewed the emergent themes in depth, cross-referencing sources to determine final themes and patterns.

## 3. Results

### 3.1. Participant Characteristics

#### 3.1.1. Demographics

This study included a total of 105 participants across 28 focus groups with 3–4 participants per group. The total sample of participants was evenly divided by race/ethnicity (49.5% Hispanic, 50.5% Black) and gender (49.5% female, 50.5% male) but generally skewed older with a majority between the ages of 15–17 (77.1%) and fewer (22.9%) between the ages of 13–14 across groups. The study included more Experimenters (66.7%) than Susceptible Non-Triers (33.3%) by design to gather use perceptions from youth who have used CLCCs. See Table 1 for a breakdown of participant characteristics and focus groups. 

#### 3.1.2. Other Tobacco Product and Marijuana Use

**Figure 1:**
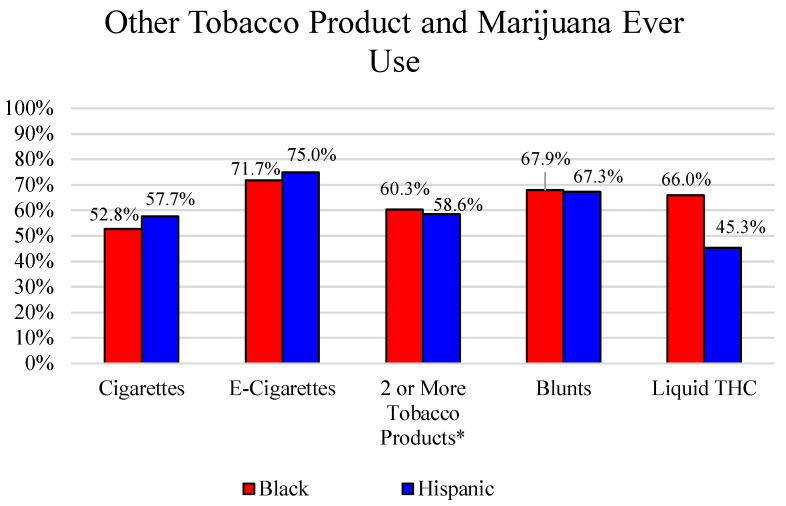
Other Tobacco and Marijuana Ever Use. * Ever use of CLCCs, Cigarettes, and E-Cigarettes was included in “2 or More Tobacco Products”.

Participants reported ever use of a variety of additional tobacco products and marijuana. A majority of Black participants reported ever use of e-cigarettes (71.7%), blunts (67.9%), and liquid THC (66.0%). For Hispanic participants, a majority reported ever use of e-cigarettes, (75.0%) and blunts (67.3%), while less than half of Hispanic participants reported ever use of Liquid THC (45.3%). Just over half of Black (52.8%) and Hispanic (57.7%) participants reported ever use of cigarettes. A majority of both Black (60.3%) and Hispanic (58.6%) participants reported using two or more tobacco products, including CLCCs. See Figure 1 for other tobacco product use by race/ethnicity.

### 3.2. CLCC Product Familiarity

**Figure 2:**
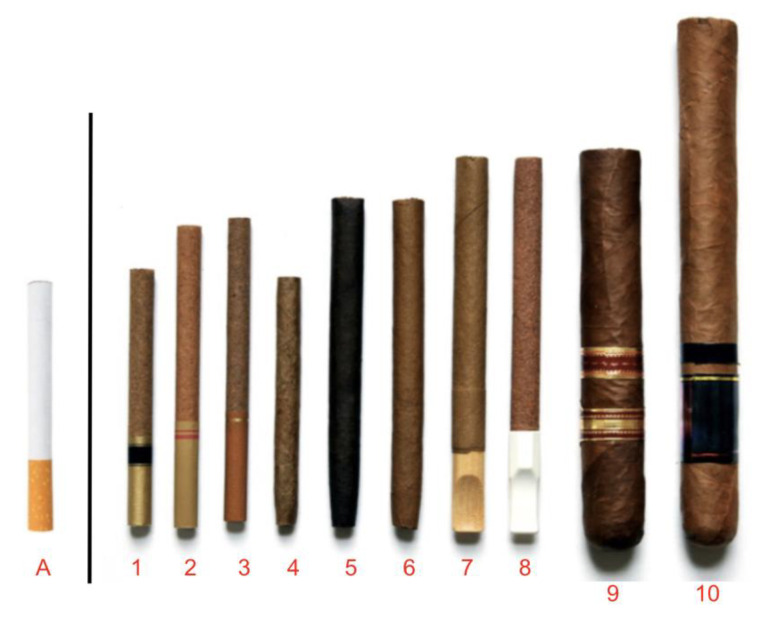
Little Cigars, Cigarillos and Cigars.

Participants were shown images derived from the FDA of various CLCC products (Figure 2) to assess familiarity and preferred terminology. The image of the cigarette (product A) was used as a point of comparison and was universally recognizable. Large cigars (products 9 and 10) were also easily recognized by all participants. By contrast, little cigars (products 1–3) were overwhelmingly unfamiliar and often thought to be a type of cigarette. Tipped cigarillos (products 7 and 8) were generally familiar, however a few susceptible non-trier youth struggled to identify these products, with one participant notably identifying one as a “whistle”. Untipped cigarillos (products 4 and 6) were the most recognized of all the CLCC products, however product 5 was much less familiar due to the darker/black color. Overall, susceptible non-trier Hispanic youth were less able to recognize and identify products in the image compared to susceptible non-trier Black youth who were widely familiar with these products.

### 3.3. Terminology

After assessing familiarity, moderators explored product terminology awareness and preferences with participants.

**Large cigars (products 9 and 10)** were universally labeled as “cigars.”

**Little cigars (products 1–3).** No participants knew the term for this product and had never heard the term “little cigars” when prompted. When asked to identify products 1–3, participants were generally unsure and often guessed on the terminology of the product, with many guessing that products 1–3 were cigarettes because of the similarity in appearance.

- ***Moderator:** What about 1, 2, 3? Any names you would call those items, those products? **Participant 1:** I would call them cigarettes, too, because they have the little—**Participant 2:** Yeah, they have the little thing that letter A has. **Participant 1:** The little colored tip at the end, so I would just call them cigarettes.*

**Cigarillos (products 4–8).** When asked, “Have you heard the term cigarillo? Which product or products would you consider a cigarillo?” participants routinely reported that “cigarillo” is not a term that is used by them or their peers. While some participants had heard or seen the term “cigarillo” or sometimes *rillo,* identifying or understanding any of the products in Image 1 as a cigarillo was rare. When asked if anyone says *rillo*, participants responded, “*I’ve heard it, but my friends don’t use that term.”; “I’ve seen it, but I’ve never heard anybody say it.”* Among those aware of the term, a handful of participants mentioned seeing the “cigarillo” term on packaging or in ads at the store, and most could not name a source (*“I don’t really ever hear anybody call it that. I just know that’s what it’s called on the package. It says cigarillo.”; “I’ve seen them [cigarillos] in stores, but I don’t really know what it means.”)*

Though youth did not understand the term “cigarillo”, they distinguished between the products that are officially considered tipped cigarillos and untipped cigarillos based on appearance. Tipped cigarillos (products 7 and 8) were often described by the brand name Black & Mild, or sometimes just called *Blacks*. However, terminology varied by location, race/ethnicity and user status. By location, the term *rillo* was used organically by only a handful of youth in the Southeast/Atlanta region and Midwest/Chicago region and was confusing or unfamiliar to most other youth when probed about the term. Youth from Midwest/Chicago region were more likely to call products 7 and 8 *Blacks.* Some Hispanic participants were familiar with the term *cigarrillo* as this is the Spanish word for cigarette, but not with the term cigarillo which *“Sounds like the Spanish pronunciation of it.”*

Untipped cigarillos (products 4 and 6) were most commonly referred to as “blunts”. Participants reported that they called untipped cigarillos “blunts” because it describes the most common way cigarillos are used by youth (“*I want to say another thing people would call [products 4 and 6] is blunts.”; “Four and 6 are blunts.”)* Aside from the term “blunt”, brand names were commonly used descriptors. Backwoods was the most commonly referenced brand and was, at times, used as the generic term to describe untipped cigarillos. Dutch Masters (*Dutches*) and then Swisher Sweets (*Swishers*) were also common brands. Other brands mentioned included: Bluntville, Game, White Owl, Jackpot, and Optimo. There was some variation by location, where some participants from the District of Columbia/Northeast region termed products 4 and 6 as *sheets and funnels* (which is a marijuana rolling style using the Bambu brand’s rolling papers known as *sheets* and Funnels brand large tobacco leaf known as *funnels*). There were also a small number of participants across locations that used the term *wrap* or *leaf* and referenced brands such as Grabba Leaf and Fronto Leaf in reference to the tobacco wrap or shell that can be purchased separately to create blunts.

### 3.4. Tobacco v. Marijuana Products

Tipped cigarillos, little cigars, and cigars were perceived by youth to be used for tobacco consumption only. These products were described as products that are used as sold and not modified for the use with marijuana. Tobacco products (e.g., cigarettes, tipped cigarillos, little cigars, and cigars) were quickly identified as harmful and undesirable (“*That’s a cancer stick. I think that’s why most people don’t have cigarettes because you could get cancer from it. I think everybody knowing that doesn’t want to smoke cigarettes.”)*

Participants not only called untipped cigarillos “blunts”, but through discussions, made clear that this was the only intended use they could imagine for these products. In all Experimenter groups and among most Susceptible Non-Trier groups, participants described in detail the process of removing tobacco from the inside of an untipped cigarillo (products 4 and 6) and inserting marijuana to make a blunt.

- ***Moderator:** After people buy it at the store, what do they do with them? **Participant:** When you pick it out the package, you split it down the middle and take the tobacco guts out of it. You can lick it down, put water on it, and then you put the weed inside of it, spread it out, and you can roll it back up.*

When asked if modified untipped cigarillos were a tobacco product, youth said that modified untipped cigarillos are not considered a tobacco product because all the loose tobacco is removed. Additionally, most youth believed only the “filling” of an untipped cigarillo contained tobacco.

- ***Participant:** The leaf is natural. It doesn’t have any nicotine. When you open up a blunt, what’s inside a blunt [is] the tobacco. The leaf is—I forgot what kind of leaf it is, but it’s just natural. There’s no chemicals added to it or anything. The one thing that does have the chemicals is the tobacco, but other than that, when you open it up, you just take it out.*

Many youth were unaware that the wrap or paper also contained tobacco and nicotine.

- ***Moderator:** Is that because once we put the weed in it, do we even feel like it’s still a tobacco product? Do we feel like it’s still tobacco? **Participant 1:** No, not at all. **Participant 2:** No.*

In many instances, youth did not know that untipped cigarillos were used for tobacco only. They perceived that blunt use is what untipped cigarillos are made for.

- ***Participant:** If it were a blunt it doesn’t matter if it has pot or tobacco in it, it would still be a blunt.*
- ***Moderator:** What do they call it [products 4 and 6] before adding the weed? **Participant 1:** Still a blunt. **Participant 2:** Yeah, they just call it blunt.*
- ***Moderator:** What about when you break down a Backwoods or you break down a blunt, what do you call it before you put the weed in it and the tobacco is out of it? **Participant:** I call it the same thing. I still call it a blunt.*

While youth perceived that using tobacco in any form was harmful, they cited fewer to no health risks for blunts which were not perceived to be a tobacco product. When youth were told that a blunt wrap contained tobacco and nicotine, they still perceived blunts to be safer than traditional tobacco products due to the belief that marijuana is “natural” and therefore safer than tobacco.

### 3.5. CLCC Product Users

In addition to distinct terminology, uses and varied harm perceptions, participants also distinguished among CLCC products that were used by youth versus adults/older people.

**Cigars.** Participants described cigars as something used by older people (30s, 40s, 50s) and not a product used by them or their peers. Additionally, youth associate cigars as a product used for celebration, but something that is not used daily *(“People sometimes, adults or whatever...they have a cigar every once in a while, when something good happens or a big party or something.”)*

**Little cigars.** Little cigars were not known by participants and therefore participants made guesses that little cigars were used by adults/older people because they resembled cigarettes which were perceived to be used by adults/older people.

**Tipped Cigarillos.** According to participants, tipped cigarillos are used by older people they knew, where “older” meant age 20+ *(“Me and my friends make a joke about people who smoke Black & Milds are older people.”; “They’re for old heads.”).* Black youth mentioned use of tipped cigarillos by older family and friends more often than Hispanic youth.

**Untipped Cigarillos.** Participants widely characterized untipped cigarillos, specifically modified as blunts, as a product that is commonly used among youth particularly in social settings.

- ***Participant:** [At a kickback], we would just be chillin’, listening to music, eating food of course, and I don’t know if I should say this, but smoking weed, I guess. We’ll pass three or four blunts around, depending on how many people around.*
- ***Participant:** When we go hang out after school, that’s what we go do. We go eat, or we go to someone’s house, and we smoke.*

Participants were widely unaware of anyone their age using untipped cigarillos unless they were modified.

## 4. Discussion

The current study points to an overall disconnect in both the terminology and intended use of CLCC products between users of these products and those conducting research around CLCCs or developing prevention programs and messaging. Findings reveal that youth generally sort CLCC products into four subgroups: (1) Blunts (or untipped cigarillos referred to by specific brand names e.g., *Dutches, Backwoods, Swisher Sweets*), that are considered a product that is modified for use with marijuana; (2) Black and Milds, tipped cigarillos that are considered a tobacco product; (3) Cigarettes (little cigar products), a tobacco product that is unfamiliar to youth; and (4) Cigars, referring to large cigars, a tobacco product.

In this study we classified youth as either Experimenters or Susceptible Non-Triers of CLCC products, defined as “cigars, cigarillos, or little cigars (like Black and Milds, Swisher Sweets, or Dutch Masters).” However, throughout the focus groups, it rapidly became clear that among Black and Hispanic youth use and susceptibility to CLCCs was focused on one product: blunts. Tipped cigarillos and little cigars were either unfamiliar or grouped as products for adults, while untipped cigarillos, understood as a modified product (a blunt), were the only CLCC product that was widely recognized and used among these youth. Even among Susceptible Non-Trier Hispanic youth who were less likely to be familiar with CLCC products overall compared to Black youth, untipped cigarillo products (blunts) were still the most recognizable. Among both Black and Hispanic youth terminology across the CLCC products was consistent, with the term “blunt” widely known and accepted.

The pervasive association of untipped cigarillos as blunts, or a product whose purpose is to be modified with marijuana, prevented the use of shared terminology for the research team and youth during subsequent discussions around the harms of CLCC products. Given lack of general familiarity with the term cigarillo, moderators tested various terms to convey the idea of an untipped cigarillo at least conceptually, including “cigarillo without marijuana,” “tobacco-only blunts” or a “Backwoods with tobacco,” to better understand use of these products “as sold/with tobacco.” However, youth exhibited strong cognitive dissonance with the idea of using an untipped cigarillo “as sold.”

These findings have important implications for public health messaging around the harms of CLCC products. In keeping with previous literature, participants in this study perceived untipped cigarillos (blunts) as less harmful than other combustible tobacco products. In this study, most CLCC experimenters did not consider untipped cigarillos to be a tobacco product, many were not aware that a cigarillo wrapper contains tobacco or nicotine, and/or many believed the modification process (replacing the tobacco filling with marijuana) made the product safer. This is in line with Kong and colleagues [10], who found that youth blunt users believed that the wrapper did not contain nicotine and therefore that they could not become addicted and did not consider themselves to be tobacco users. In another qualitative study among young adult Black males, participants stated that the removal of tobacco from untipped cigarillos for blunt use was specifically what made the product less harmful [15]. These perceptions pose a significant challenge but also an important opportunity for awareness messaging around tobacco and nicotine content in CLCC product wrappers.

Additionally, while this study provides insights into use patterns among youth, a deeper understanding is needed into the varied use and interrelationship among CLCC products (and other tobacco products) among youth. Prior research among youth and young adults has shown that simultaneous use and co-administration of CLCC products is common [12,22]. This includes behaviors such as “chasing,” i.e., using a tobacco product immediately after marijuana, and substitution, i.e., using a CLCC product when marijuana is not available [22]. These behaviors may also help to explain why some participants in the current study reported ever use of CLCC products WITHOUT marijuana and yet considered themselves blunt users.

Overall, these findings underscore a need to rethink measurement of youth CLCC use and susceptibility to better align with the terminology and use patterns of youth most at-risk. Whenever possible, data collection instruments seeking to measure use and susceptibility should measure little cigar and cigarillo use using distinct questions with terminology recognized by youth (e.g., brand names). While some current national studies such as the NYTS do offer this level of granularity, cigarillo and little cigar use are measured as a branching question where participants are first asked about CLCC use overall. Given that youth do not group these products together, along with their overall low familiarity with little cigars, this type of combined metric may lead to confusion and therefore less accurate data. Moreover, given that those who use blunts consider them to be marijuana products, blunt users may be missed on this type of branching question altogether. Further distinguishing between tipped and untipped cigarillos, where possible, would improve measurement accuracy given the pervasive exposure to and use of untipped cigarillos among this demographic. In terms of terminology, data instruments targeting youth should couple researcher terminology with terms used by youth along with examples of popular brands. For example, instruments should use language such as “tipped cigarillos are products with a plastic or wooden tip such as a Black and Mild.” Using imagery of CLCC products can provide further clarity and alignment on the unit of measurement. Finally, because modification with marijuana is so pervasive among youth, metrics should be explicit about whether use of the product of interest should be considered “WITH marijuana (such as a blunt)” or “WITHOUT marijuana.”

This study has limitations. Foremost, this is a qualitative research study and therefore findings are not generalizable and should be further explored quantitatively. Furthermore, the recruitment for this study focused on Black and Hispanic youth predominantly from “city hubs” and as such respondents were disproportionately from urban environments. This sample therefore cannot be considered a representative sample of Black and Hispanic CLCC experimenting and susceptible youth across U.S. regions and settings.

## 5. Conclusions

The current study, through a series of focus groups, investigated the terminology and use of CLCC products among Black and Hispanic youth. Results show that these youth are most familiar with cigarillo products and differentiate between tipped and untipped cigarillos in terms of both terminology and use patterns. Understanding the nuances of the language and use patterns of CLCC products among youth is critical to ensuring both the accuracy of CLCC measurement as well as the impact of public health strategies and messaging. Ultimately improving the specificity of CLCC terminology will enable researchers and public health practitioners to characterize prevalence patterns of youth CLCC susceptibility and use as well as develop more effective prevention messaging leading to the design of more impactful public health strategies and programs.

## Figures and Tables

**Table 1:** Sample Characteristics.

Demographics (Total *n* = 105)		Total *n*	%	Number of Groups
**Age**	13–14	24	22.9%	--
	15–17	81	77.1%	--
**Gender**	Female	52	49.5%	--
	Male	53	50.5%	--
**Race/Ethnicity**	Hispanic	52	49.5%	14
	Black	53	50.5%	14
**Location**	West/Los Angeles Region	27	25.7%	7
	Southeast/Atlanta Region	24	22.9%	7
	Midwest/Chicago Region	26	24.8%	7
	Northeast/District of Columbia Region	28	26.7%	7
**CLCC Use Status**	Experimenter	70	66.7%	19
	Susceptible Non-Trier	35	33.3%	9

## Data Availability

The data presented in this study are available on request from the corresponding author. The data are not publicly available due to participant confidentiality.
